# Toothbrushing ability, caries burden, and oral health–related quality of life among stunted preschool children in Bandung-Indonesia

**DOI:** 10.1186/s12903-025-07307-1

**Published:** 2025-12-29

**Authors:** Arlette Suzy Setiawan, Irene Tanesha Atmadja, Prima Andisetyanto, Ratna Indriyanti, Netty Suryanti, Neti Juniarti, Laili Rahayuwati

**Affiliations:** 1 Department of Pediatric Dentistry, Faculty of Dentistry, Universitas Padjadjaran, Bandung, Indonesia; 2 Dentistry Education Program, Faculty of Dentistry, Universitas Padjadjaran, Bandung, Indonesia; 3 Department of Community Dentistry, Faculty of Dentistry, Universitas Padjadjaran, Bandung, Indonesia; 4 Faculty of Nursing, Universitas Padjadjaran, Bandung, Indonesia

**Keywords:** Stunting, Children’s motor skills, Tooth brushing skills

## Abstract

**Background:**

Stunting remains a significant public health challenge in Indonesia and may affect children’s neuromotor development, including fine motor coordination required for effective toothbrushing. Impaired oral hygiene behaviors in stunted children may contribute to increased caries burden and reduced oral health–related quality of life (OHRQoL).

**Objective:**

To investigate the relationship between toothbrushing ability, caries burden, and OHRQoL among stunted and non-stunted preschool children in Bandung, Indonesia.

**Methods:**

A cross-sectional study was conducted among 554 children aged 1–5 years selected through consecutive sampling from identified stunting loci. Clinical assessments of caries (def-t index) and oral hygiene (Debris Index from OHI-S) were conducted by calibrated dentists. Toothbrushing ability was assessed using a structured checklist, and OHRQoL was assessed using the validated Indonesian version of the Parental-Caregiver Perception Questionnaire (P-CPQ). Statistical analyses included descriptive statistics and chi-square tests to examine bivariate associations, with linear-by-linear association tests for ordinal variables.

**Results:**

Nutritional status was significantly associated with toothbrushing ability (*p* = 0.034) and OHRQoL (*p* < 0.001), but not with caries burden (*p* = 0.924). Toothbrushing ability was significantly associated with caries burden (*p* < 0.001) and oral hygiene (*p* < 0.001). Oral hygiene was also associated with caries burden (*p* < 0.001). Brushing frequency (*p* = 0.048) and technique (*p* = 0.027) differed significantly by nutritional status, whereas brushing duration, parental assistance, and grip type did not. No significant associations were observed between caries burden, oral hygiene, toothbrushing ability, and OHRQoL, likely due to the highly skewed distribution of OHRQoL categories, with 97.5% classified as “very low impact.”

**Conclusion:**

Stunted children demonstrated poorer toothbrushing ability compared to their non-stunted peers, and caries burden was closely related to oral hygiene and brushing practices. Nutritional status was associated with OHRQoL, although other variables showed no significant relationship due to limited variability in OHRQoL scores. Integrating oral health promotion with nutrition improvement programs may support better oral and general health outcomes among nutritionally vulnerable populations.

**Supplementary Information:**

The online version contains supplementary material available at 10.1186/s12903-025-07307-1.

## Introduction

Stunting continues to pose a significant challenge in developing countries, with Indonesia being no exception and exhibiting a disturbingly high prevalence [1]. According to the findings from the 2021 Indonesian Nutrition Status Study, the prevalence of stunting among children under five years of age in Indonesia was 24.4%. This issue is particularly pronounced in West Java Province, where stunting is slightly greater at 24.5%, within the medium range [2]. Stunting is a tangible manifestation of the intersection between nutritional deficiencies and infections, manifesting both during pregnancy and in the early stages of a child’s life [3]. The inadequate intake of vital nutrients during prenatal and initial growth phases profoundly influences the development of a child’s brain. This can give rise to a spectrum of neurological abnormalities, ultimately impacting crucial domains such as motor skills, cognition, language proficiency, and socioemotional aptitudes and even potentially leading to mental retardation [4, 5]. Notably, a study conducted by Manggala et al. in 2018 underscored that children experiencing stunting in Indonesia often exhibit characteristics of social withdrawal and diminished cognitive and motor responses [6]. 

Interestingly, a distinct correlation exists between the decline in motor function among stunted children without congenital abnormalities and the delayed maturation of muscle function in the triceps muscles [7]. This correlation is particularly significant since proficient oral hygiene, including tooth brushing, relies heavily on robust motor skills [8]. Effective tooth brushing hinges on various factors, including appropriate tools and techniques and the frequency and duration of these oral care practices [9–11].

Furthermore, studies have consistently demonstrated an intriguing association between dental caries and tooth brushing habits. The crux of this relationship lies in the continuous removal of plaque accumulation achieved through regular tooth brushing, which subsequently reduces the risk of dental caries [12, 13]. Given the mounting evidence of diminished motor and cognitive abilities among stunted children compared to their normally developing peers, this study endeavours to provide a comprehensive overview of the tooth-brushing capabilities exhibited by stunted children. This examination encompasses crucial aspects such as the frequency of tooth brushing, the duration of these oral care practices, and the specific tooth brushing techniques employed.

Stunting, a key target of Sustainable Development Goals (SDG 2 and SDG 3), remains a major public health issue worldwide and is associated with long-term health and developmental consequences. In Indonesia, the prevalence remains among the highest in Southeast Asia. Fine motor development is critical for performing effective oral hygiene, particularly toothbrushing, which requires adequate grip, control, and movement coordination. Neurodevelopmental delays in stunted children may limit these skills, potentially leading to poorer oral health outcomes.

Despite these connections, few studies have examined the interplay between toothbrushing ability, caries burden, and OHRQoL in preschool children with stunting. This study aims to fill that gap by testing the hypothesis that stunted children have poorer toothbrushing ability, higher caries burden, and lower OHRQoL, with caries burden being the strongest predictor.

## Materials and methods

### Study design and setting

This study employed a cross-sectional observational design to examine the association between toothbrushing ability, caries burden, and oral health-related quality of life (OHRQoL) among stunted preschool children in Indonesia. The research was conducted between June 2023 and May 2024 in Bandung City, Indonesia.

### Participants and sampling

**Fig. 1:**
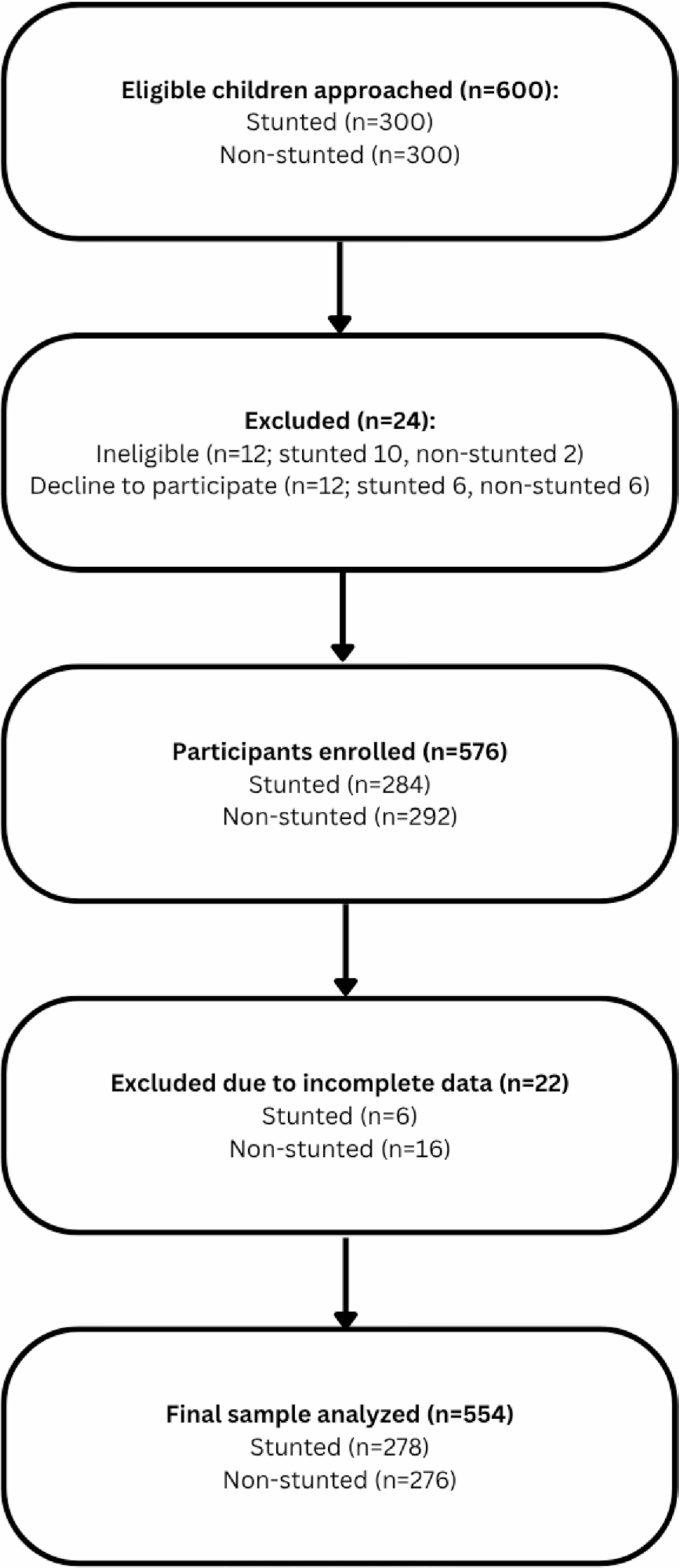
Flowchart participant recruitment

Eligible participants were preschool children aged 1–5 years, accompanied by a parent or primary caregiver, and who had no systemic condition affecting oral health other than stunting. A sex and age matched children as control group also recruited from the same demographic background. A total of 600 eligible were approached, of whom 576 agreed to participate, yielding a 96% response rate. From these, 554 participants (278 stunted and 276 non-stunted) had complete data and were included in the analysis. Participants were recruited using a consecutive sampling approach from community health centers. The participant recruitment flowchart presented in Fig. 1.

### Nutritional status classification and validation

Nutritional status was determined based on the World Health Organization (WHO) Child Growth Standards. Stunting was defined as a height-for-age Z-score below − 2 standard deviations (SD), while children with a Z-score ≥ − 2 SD were classified as non-stunted. Anthropometric measurements (height and age) were obtained from the Bandung City Health Office surveillance database and were validated through direct measurements at Posyandu (community health posts) by trained health workers using standardized equipment and procedures. The WHO Anthro software (version 3.2.2) was used to calculate Z-scores for height-for-age.

### Sample size calculation

The minimum sample size for this cross-sectional study was calculated usiang a formula for comparing two proportions, based on the prevalence of poor oral health-related quality of life (OHRQoL) among stunted and non-stunted preschool children in a previous Indonesioan study [14]. n that study, 45% of stunted children and 28% of non-stunted children were reported to have poor OHRQoL (P₁ = 0.45, P₂ = 0.28).

Using a two-sided α of 0.05 and 80% power, the sample size was calculated as:

$$\:n=\frac{({Z}_{1-\alpha\:/2}\sqrt{2P(1-P)}+{Z}_{1-\beta\:}\sqrt{{P}_{1}(1-{P}_{1})+{P}_{2}(1-{P}_{2})}{)}^{2}}{({P}_{1}-{P}_{2}{)}^{2}}$$

where $$\:P=\frac{({P}_{1}+{P}_{2})}{2}$$.

This yielded a minimum sample size of 240 children per group (480 total). Allowing for a 10% non-response rate, the target sample size was 528 children. The final analyzed sample consisted of 278 stunted and 276 non-stunted children, exceeding the minimum requirement.

### Selection of participants

Non-stunted children were recruited from the same geographical areas as the stunted children and matched by age (± 3 months) and sex to minimize confounding. Children with diagnosed developmental, neurological, or physical disabilities were excluded. The final analyzed sample comprised 278 stunted children and 276 non stunted due to incomplete data and participant withdrawal.

### Ethics approval and consent

The study was approved by the Universitas Padjadjaran Research Ethics Committee (approval no. 517/UN6.KEP/EC/2023) and subsequently renewed (Approval No. 855/UN6.KEP/EC/2024) as the research period exceeded one year, following the Declaration of Helsinki Written informed consent was obtained from parents or primary caregivers prior to participation.

### Clinical oral examination

Two trained and calibrated pediatric dentists performed all oral examinations to assess dental caries (deft index) and oral hygiene (OHI-S debris index). Caries burden was operationalized as the percentage of decayed, extracted, and filled primary teeth relative to the total number of erupted teeth (def-t%). This percentage accommodates variations in eruption patterns in preschool-aged children [15, 16]. Categories of caries burden (high, moderate, low, none) were adapted from Indriyanti et al. (2021) [14], where high corresponds to def-t% >51%, moderate 26–50%, low *≤* 25%, and none 0%.

Oral hygiene status in children with primary dentition was assessed using the Simplified Oral Hygiene Index (OHI-S) by Greene and Vermillion (1964), focusing only on the Debris Index (DI) component. Because the participants were preschool children with primary teeth, the index teeth were adapted according to the eruption status of the primary dentition. The buccal and lingual surfaces of representative primary molars and incisors were examined.

Each tooth surface was scored from 0 to 3 based on the extent of soft debris (plaque) accumulation:

- 0 = No debris or stain present.
- 1 = Soft debris covering not more than one third of the tooth surface.
- 2 = Soft debris covering more than one third but not more than two thirds of the tooth surface.
- 3 = Soft debris covering more than two thirds of the tooth surface.

The Debris Index score was calculated by summing the scores for all examined surfaces and dividing by the total number of surfaces assessed, producing a mean DI score between 0.0 and 3.0.

The oral hygiene status was then categorized as follows:

- Good (0.0–0.6).
- Fair (0.7–1.8).
- Poor (1.9–3.0).

This classification reflects the level of plaque accumulation on primary teeth, with lower scores indicating better oral hygiene.

Calibration was conducted before data collection, with intra- and inter-examiner reliability measured using Cohen’s kappa (> 0.85 for both indices). The deft percentage was calculated as the proportion of decayed, extracted, and filled primary teeth relative to the total number of erupted primary teeth, accommodating differences in eruption stage among preschoolers.

### Assessment of toothbrushing ability

Toothbrushing ability was evaluated using a checklist adapted from a validated instrument. Adaptation involved forward–backward translation into Bahasa Indonesia, expert panel review (pediatric dentists and public health specialists). The checklist assessed grip type, brushing control, movement coordination, and brushing duration. Observations were conducted directly by trained examiners, supplemented with caregiver confirmation when needed.

Prior to the main data collection, the adapted toothbrushing ability checklist was pretested among 20 preschool children to assess clarity, feasibility, and variability of responses. The pretest aimed to ensure that parents and observers could understand the wording and sequence of the items, and that children’s responses covered the expected range. Based on the pretest, minor wording adjustments were made to improve parental comprehension, and some checklist items were reordered for smoother administration. As the pretest primarily served to refine translation and content, no inferential statistical analyses were conducted. Descriptive summaries were used to examine response distributions and confirm item variability. Inter-rater reliability testing was not performed at this stage, which is acknowledged as a study limitation.

Toothbrushing ability was measured by a structured questionnaire and an observation checklist, which comprises five indicators: (1) frequency of toothbrushing, (2) parental assistance during toothbrushing, (3) duration of toothbrushing, (4) toothbrushing technique, and (5) toothbrush grip type [8]. The scoring criteria for each indicator are detailed in Supplement 1. Each item was scored 0 to 3 with total scores in the range from 0 to 15, then it was grouped into categories as follows:

- Excellent (13–15 points): The child brushes with parental assitance, uses proper technique and grip, and brushes for an appropriate duration with adequate frequency.
- Good (10–12 points): The child demonstrates adequate brushing ability with some areas needing minor improvement.
- Fair (7–9 points): The child requires regular supervision and assistance to improve brushing skills.
- Poor (≤ 6 points): The child exhibits low brushing ability and requires significant guidance and oral health intervention.

### Assessment of oral health–related quality of life (OHRQoL)

OHRQoL was measured using the Preschool Child Perceptions Questionnaire (P-CPQ), validated for Indonesian preschool populations. This instrument was chosen due to its comprehensive domain coverage and comparability to international research. Alternative tools such as SOHO-5 or COHIP were considered but excluded due to lack of local validation. Questionnaires were completed by the primary caregiver (94.6% mothers) at community health centers, with trained enumerators providing clarification when necessary.

The OHRQoL was evaluated by applying the short-form Parental-Caregiver Perception Questionnaire (P-CPQ), which has 16 items that fall into four domains: oral symptoms, functional limitations, emotional well-being, and social well-being [17]. Each item was rated on a 5-point Likert scale: 0 = never, 1 = once or twice, 2 = sometimes, 3 = often and 4 = every day or almost every day. Responses marked “don’t know” were excluded. The total score ranged from 0 to 64; higher scores indicate worse perceived quality of life. Scores were categorized into the following groups: Very low impact (0–16), low impact (17–32), moderate impact (33–48) and high impact (49–64). The Indonesian version of the P-CPQ, used in this study, has been validated previously with a Cronbach’s alpha of 0.843 [14, 18]. 

### Sociodemographic data and potential confounders

Parental education, household income, and access to dental care were collected through structured interviews and included as potential confounders in multivariable models.

### Statistical analysis

Data normality was assessed using the Shapiro–Wilk test, and homogeneity of variances was evaluated using Levene’s test. Descriptive statistics were calculated for all variables. Bivariate associations between categorical variables (e.g., toothbrushing ability, nutritional status, caries burden, oral hygiene, and OHRQoL) were examined using chi-square tests. For ordinal variables, linear-by-linear association tests were additionally conducted to assess potential trends. Independent t-tests and one-way ANOVA were used for continuous variables, with Tukey’s HSD post hoc tests applied when appropriate. Statistical significance was set at *p* < 0.05. All analyses were performed using IBM SPSS Statistics, version 23 (IBM Corp., Armonk, NY, USA).

## Results

A total of 554 children aged 1–5 years participated in this study (mean age overall = 3.3 years, SD = 1.2), with the largest proportion aged 3 and 4 years (30.0%). Other age groups (2 and 5 years) were relatively evenly distributed (16.8–23.3%). The gender distribution was balanced (50.5% male, 49.5% female). Nutritional status classification showed 50.2% stunted and 49.8% non-stunted children.

The recruitment process is illustrated in Supplementary Fig. S1, following STROBE guidelines. A total of 600 eligible children were approached, of whom 576 agreed to participate, yielding a response rate of 96%.

Table 1 presents the sociodemographic and oral health characteristics of respondents. All respondents were mothers. Most were aged 26–35 years (50%) and were either housewives or employees (private sector or government) 48.9% each. Educational attainment was predominantly senior high school (80.58%), with a small proportion holding a bachelor’s (0.6%) or master’s degree (0.6%).

Regarding oral health behaviors, 31.0% of children had “good” toothbrushing ability, 48.6% “fair,” 13.4% “poor,” and 7.0% “excellent.” Oral health–related quality of life (OHRQoL) was categorized as “very low impact” in 97.5% and “low impact” in 2.5% of children. The term “caries burden” is used consistently to refer the percentage of decayed, extracted, and filled primary teeth relative to the total number of erupted teeth (def-t%). Caries burden was high in 15.3% of children, moderate in 46.6%, low in 34.1%, and none in 4.0%. The Oral Hygiene Index-Simplified (OHI-S) classification showed 47.5% fair oral hygiene, 21.7% good, and 30.9% poor.

**Table 1:** Frequency distribution of respondent characteristics (*n* = 554)

Variable	Category	Frequency (*n*)	Percentage (%)
Gender			
- Parent	Mother	544	100
	Father	0	0
- Child	Male	280	50.5
	Female	274	49.5
Age			
- Parent	≤ 25	96	17.7
	26–35	272	50
	36–45	153	28.1
	> 45	23	4.2
- Child	1	0	0
	2	93	16.8
	3	166	30.0
	4	166	30.0
	5	129	23.3
Parental education	Elementary school	16	2.9
	Junior high school	30	5.5
	Senior high school	490	90.1
	Diploma	2	0.3
	Bachelor	3	0.6
	Master	3	0.6
	Doctoral	0	0
Parental occupation	Housewife	266	48.9
	Self-employed	12	4.3
	Employee	266	48.9
Child’s Nutritional Status	Stunting	278	50.2
	Non-stunting	276	49.8
Toothbrushing ability	Poor	74	13.4
	Fair	269	48.6
	Good	172	31.0
	Excellent	39	7.0
OHRQoL	low Impact	14	2.5
	Very low impact	540	97.5
Caries burden (def-t%)	High impact	85	15.3
	Moderate impact	258	46.6
	Low impact	189	34.1
	No impact	22	4.0
Oral hygiene (OHI-S)	Good	120	21.7
	Fair	263	47.5
	Poor	171	30.9

*Bivariate associations* between key study variables are presented in Table 2. Toothbrushing ability was significantly associated with caries burden (χ² = 43.65, df = 9, *p* < 0.001), nutritional status (χ² = 8.67, df = 3, *p* = 0.034), and oral hygiene (OHI-S) (χ² = 268.10, df = 3, *p* < 0.001). Additionally, oral hygiene was strongly associated with caries burden (χ² = 50.61, df = 6, *p* < 0.001), indicating that poorer oral hygiene corresponded to higher caries impact. In contrast, no significant association was observed between nutritional status and caries burden (χ² = 0.48, df = 3, *p* = 0.924).

With respect to OHRQoL, nutritional status was significantly associated with OHRQoL categories (χ² = 14.68, df = 1, *p* < 0.001), with stunted children more likely to experience greater impacts on oral health–related quality of life compared to their non-stunted peers. Conversely, toothbrushing ability (χ² = 3.26, df = 3, *p* = 0.353), caries burden (χ² = 5.47, df = 3, *p* = 0.141), and oral hygiene (χ² = 4.21, df = 3, *p* = 0.122) were not significantly associated with OHRQoL.

The lack of significant associations for these variables is likely influenced by the highly skewed distribution of OHRQoL scores, with 97.5% of participants categorized as “very low impact,” limiting the sensitivity of statistical tests to detect group differences.

Multivariable analysis was not performed due to the highly skewed distribution of OHRQoL categories, with 97.5% of participants classified as “very low impact.” This distribution violated key assumptions for ordinal logistic regression and limited the sensitivity of multivariable modeling. Therefore, the analysis focused on descriptive and bivariate associations to explore relationships between nutritional status, caries burden, toothbrushing ability, oral hygiene, and OHRQoL.

Interaction analysis between nutritional status and toothbrushing ability on OHRQoL domain scores was not further pursued. The distribution of OHRQoL responses was highly skewed, with the vast majority of children classified in the “very low impact” category, limiting the appropriateness and interpretability of two-way ANOVA for domain-specific analyses. Therefore, interaction effects were not retained in the final analysis.

*Item-level analysis* (Table 3.) showed significant differences between stunted and non-stunted children in 12 of the 16 P-CPQ items (*p* < 0.05), including oral pain, gingival bleeding, halitosis, eating difficulty, sleep disturbance, frustration, and absenteeism. No differences were found for anxiety, smile refusal, or learning difficulty. The “play refusal” item showed zero variance (all responses identical) and was excluded from statistical testing.

For toothbrushing behaviors, nutritional status was significantly associated with brushing frequency (*p* = 0.048) and technique (*p* = 0.027), with non-stunted children more likely to brush ≥ 2× daily and to use proper techniques (e.g., circular motion). Brushing duration, parental assistance, and grip type were not significantly associated with nutritional status. Linear-by-linear association tests confirmed the trends for brushing frequency and technique (*p* < 0.05).

**Table 2:** Relationship between variables of tooth brushing ability, nutritional status, OHRQoL, OHI-S, and caries burden (*n* = 554)

Variable Pairs	Chi-Square	df	*p*-value	Remarks
Toothbrushing ability x caries burden	43.649	9	< 0.001	Significant
Nutritional status x caries burden	0.476	9	0.924	Non-ignificant
Oral hygiene x caries burden	50.613	6	< 0.001	Significant
Toothbrushing ability x nutritional status	8.8670	3	0.034	Significant
Toothbrushing ability x oral hygiene	268.099	3	< 0.001	Significant
Toothbrushing ability x OHRQoL	3.263	3	0.35.3	Non-significant
Nutritional status x OHRQoL	14.677	1	< 0.001	Significant
Caries burden x OHRQoL	4.465	3	0.141	Non-significant
Oral hygiene x OHRQoL	4.211	32	0.122	Non-significant

**Table 3:** Parental responses to the oral health-related quality of life and toothbrushing ability questionnaires

Questionnaire	Item	Dominant response		X^2^	p-value	Interpretation
		Stunting	Non-stunting			
Oral Health Related Quality of Life	Oral pain	Never	Occasionally	131.28	0.000	Significant
	Gingival bleeding	Never	Never	131.81	0.008	Significant
	Halitosis	Never	Never	16.801	0.001	Significant
	Food impaction	Occasionally	Occasionally	78.419	0.000	Significant
	Eating difficulty	Never	Never	58.509	0.000	Significant
	Slow eating	Never	Never	58.773	0.000	Significant
	Sensitive tooth	Never	Never	35.695	0.000	Significant
	Sleep depriviation	Never	Never	30.382	0.000	Significant
	Frustration	Never	Never	73.031	0.000	Significant
	Embarrassment	Never	Never	17.412	0.000	Significant
	Anxiety and Fear	Never	Never	5.091	0.078	Non-significant
	Speak reluctant	Never	Never	10.111	0.006	Significant
	Smile refusal	Never	Never	4.000	0.135	Non-significant
	Absenteeism	Never	Never	38.696	0.000	Significant
	Learning difficulty	Never	Never	1.995	0.365	Non-significant
	Play refusal	Never	Never			Unable to compute
Toothbrushing ability	Toothbrushing frequency	3x/day	Never	7.926	0.048	Significant
	Parental assisstant	Effective assistance	Inconsistent assistance	0.990	0.804	Non-significant
	Toothbrushing Duration	About 1.5 min	< 1 min or not brushed	3.370	0.338	Non-significant
	Toothbrushing technique	Circular	Vertical	9.175	0.027	Significant
	Toothbrush grip	Oblique grip	Spoon	1.754	0.625	Non-significant

## Discussion

This cross-sectional study found that stunted preschool children tended to have lower toothbrushing ability and a higher burden of dental caries compared to their non-stunted peers. These findings should be interpreted as associations rather than causal relationships, given the study design. The observed patterns may reflect broader disparities in health behaviors and access to care rather than direct nutritional effects of stunting on oral health.

Although parental education and occupation were collected, they did not show significant associations with stunting in our sample. Nevertheless, previous studies consistently report that lower parental education and unstable employment are linked to higher stunting prevalence, primarily through reduced health literacy and barriers to preventive care. This context may help explain the differences we observed in toothbrushing behaviors and caries burden.

In this study, OHRQoL scores were highly skewed, with 97.5% of children categorized in the “very low impact” group. This distribution limited the ability to detect associations between oral health variables and OHRQoL, which may explain the lack of significance in several bivariate analyses. Item-level comparisons, however, revealed significant differences in 12 of 16 P-CPQ items, suggesting that specific aspects of oral health still affect the daily lives of stunted children.

Parents play a crucial role in maintaining their children’s dental and oral health [19, 20]. They can actively participate by guiding, providing understanding, reminding, and providing facilities to their children. Active parental involvement helps children develop a routine habit of maintaining dental and oral health [13]. By acquiring fundamental knowledge from their parents, children are expected to apply it independently in their daily lives [21–23]. 

In this study, the characteristics of the parents are depicted in terms of sex and age. Most respondents were aged 26–35 years, which aligns with the classification of early adulthood according to the Indonesian Ministry of Health. At this stage, individuals have mature thinking patterns and can actively contribute to their child’s well-being. Regarding occupation, most respondents are not employed and are homemakers. According to research conducted by Savita in 2020, nonworking mothers have a fivefold greater risk of having stunted children than working mothers [13]. For the highest education level, most parents had completed only junior high school [24]. According to a study by Rachman et al. in 2021, one of the risk factors for stunting in Indonesian toddlers is the parents’ education level, as it indirectly affects their healthy lifestyle pattern [25]. 

Dental and oral health can significantly impact overall quality of life. The most common dental and oral health issue found is dental caries, which is why the def-t scores of each stunted child in this study were calculated [26]. This study investigated the oral health-related quality of life (OHRQoL) of stunted children by measuring the impact of various oral diseases on their well-being from the perspective of parents/caregivers through the parental-caregiver perception questionnaire (P-CPQ) [14]. The assessment was conducted in four dimensions: oral symptoms, functional limitations, emotional well-being, and social well-being (Table 3.). As shown in the table, the overall OHRQoL of the children in all four dimensions was still considered good. This finding is consistent with a previous study on adolescents with juvenile idiopathic arthritis, which revealed no significant correlation between dental caries and OHRQoL [27]. This finding is also supported by Khawana Faker et al.‘s study, which revealed that children over 6 years have a greater chance of experiencing negative impacts on OHRQoL than children under 6. This is because dental caries in older children is usually in more advanced stages, resulting in more significant negative impacts on OHRQoL [28]. 

In the emotional and social well-being dimensions, most parents/caregivers did not perceive significant impacts on their children, possibly because their ability to express emotions is still limited. This is supported by previous research stating that children six years and older have a greater capacity to express emotions, including communicating the effects of their oral health condition on their quality of life to their parents, than toddlers [18, 28]. Regarding emotional aspects, most children in this study also tended not to feel embarrassed or anxious about their dental condition, as their peers in the surrounding environment also had similar dental conditions. Additionally, regarding social aspects, all children in this study were still of preschool age, limiting their social interactions to their home environment only.

The assessment of toothbrushing ability among stunted children is depicted in Tables 4 and 5. The first indicator in the assessment is the frequency of toothbrushing. Most children in this study brushed their teeth two or more times a day. This frequency is in line with the recommendations of the WHO and FDI, and it has also been reported in other countries where most of the population, across all age groups, brush their teeth two or more times a day. Brushing teeth twice a day is believed to reduce the prevalence of dental caries compared to brushing teeth only once daily. Additionally, similar beneficial effects of brushing teeth twice daily have been found on gingival health [28]. 

Parental assistance during brushing was common across both groups but did not differ significantly by nutritional status. This suggests that assistance alone may not be sufficient to improve oral health behaviors or outcomes if the quality of assistance (e.g., technique, supervision quality, consistency) is suboptimal. Similarly, while the horizontal brushing technique was most commonly reported, our study did not evaluate its effectiveness; previous research indicates that circular or modified Bass techniques are generally more effective for plaque removal, especially in children.

The discussion on brushing duration has been revised to include supporting references indicating that durations shorter than 2 min are associated with significantly lower plaque removal efficiency [9, 10]. Grip type was analyzed as a proxy for fine motor development, as certain grips (e.g., oblique or cylindrical) reflect more advanced motor skills. However, in our sample, grip type was not significantly associated with nutritional status, suggesting that fine motor delays—if present—may not be captured by this measure alone or may be influenced by other environmental or developmental factors.

The second indicator is the assistance provided by parents or caregivers during toothbrushing, and it was found that most children in this study still needed assistance from their parents or caregivers when brushing their teeth. Dental and oral health maintenance in children heavily relies on parents, especially mothers. Therefore, mothers need to know how to care for their children’s teeth and provide guidance on proper toothbrushing techniques. Children, especially toddlers, cannot effectively and correctly maintain oral hygiene on their own, so parents need to assist in toothbrushing until the child reaches 6 years of age and continue to supervise the procedure consistently [27].

In addition to frequency and parental assistance in toothbrushing, the duration of toothbrushing is also an important factor in plaque removal. Toothbrushing that is too short is considered one of the main causes of inadequate oral hygiene [29]. Most dental and oral health care experts recommend that the ideal toothbrushing duration be at least 2 min. However, previous research has shown that the estimated brushing time ranges from only 30 seconds to 60 seconds [30]. This is consistent with the results of this study, where most children spent approximately 1 min each time they brushed their teeth. This duration is insufficient to significantly reduce plaque, as the effect can only be achieved after 30–45 seconds of brushing per quadrant, which amounts to 2–2.5 min for all quadrants. A longer duration does not result in a significant increase in plaque reduction. Therefore, even though some children in this study brushed their teeth for 3 min, this duration is still considered inefficient [29]. 

The toothbrushing technique used is also equally important. Generally, the horizontal and circular techniques (Fones) are the two recommended methods for children. The Fones technique involves placing the toothbrush on the buccal surface of the teeth in occlusion and moving it in a circular motion from the maxillary to the mandibular gingiva with light pressure on the toothbrush head. This technique is often taught to children because it is easy to follow [31]. The horizontal technique involves placing the active surface of the toothbrush perpendicular to the tooth surface and moving it back and forth on all parts of the dental arch [32]. In this study, the most used technique was the horizontal technique. Scientific evidence from the studies conducted thus far is insufficient to conclude that one toothbrushing technique is more effective than the others, especially for children.

**Fig. 2:**
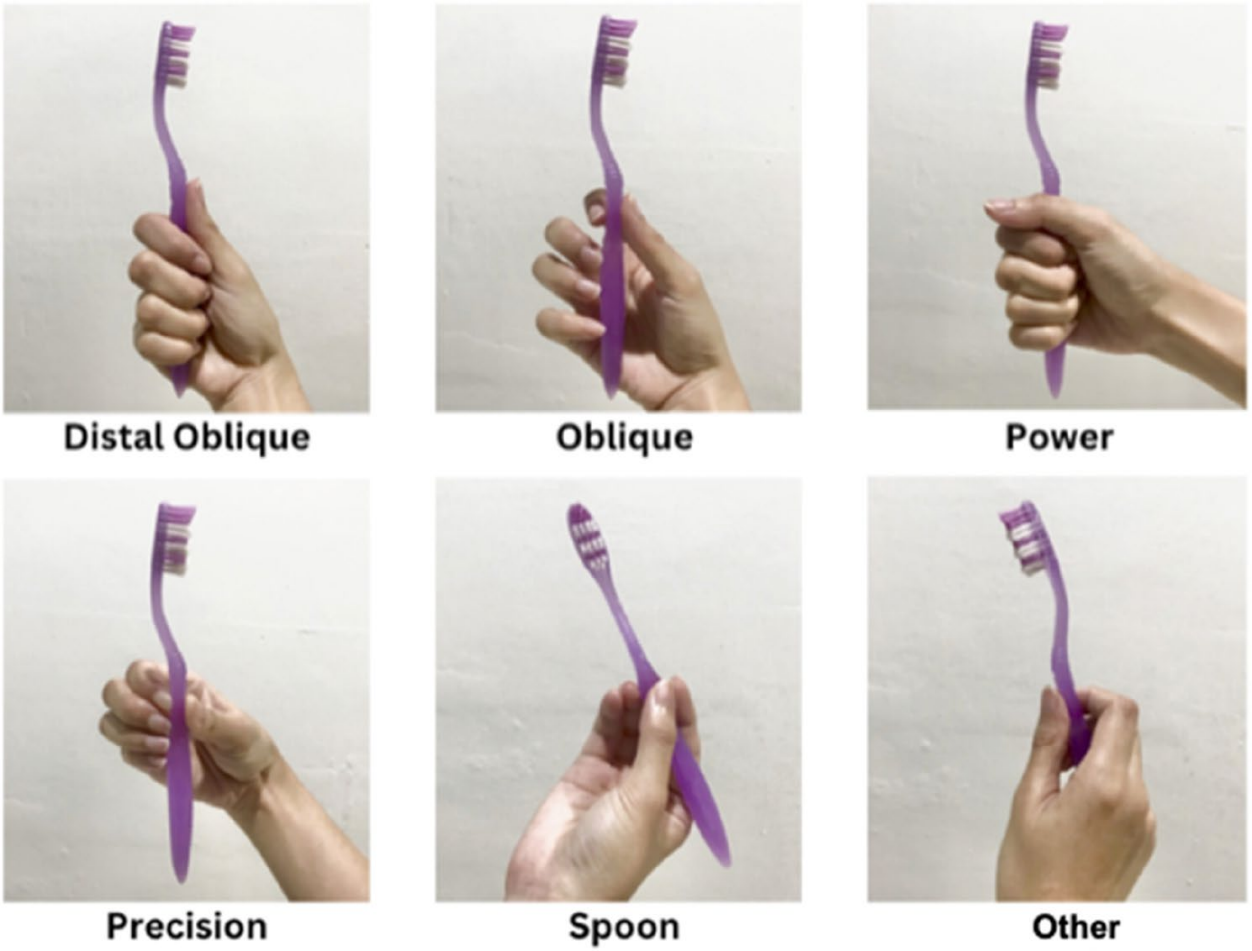
Toothbrush grip types: distal oblique, oblique, power, precision, spoon, and other

The grip tape on the toothbrush can be categorised into five types according to Beals et al., namely, distal oblique, oblique, power, spoon, precision, and ‘others’ (Fig. 2). The most frequently chosen grip type by stunted children in this study was the distal oblique. This result is consistent with research conducted by Iqra Muhammad Khan et al., Vijay Lakshmi et al., and P. Pujar with V. V. Subbareddy. In all three of these studies, grip type on the toothbrush did not significantly contribute to children’s toothbrushing ability [33–35]. Overall, 44.87% of the stunted children in this study had sufficient toothbrushing ability, 39.74% had poor ability, and only 12% had good ability.

While the integration of oral and general health programs was proposed, our discussion now includes specific strategies: (1) incorporating oral health education into national stunting reduction campaigns, (2) training community health workers to deliver both nutrition and oral hygiene counseling during routine child health visits, (3) integrating dental screening into growth monitoring programs, and (4) implementing home-based toothbrushing programs in collaboration with early childhood education centers.

Finally, although we observed associations between stunting and toothbrushing ability, these may be confounded by socioeconomic status. Our study did not adjust for SES variables such as household income, parental occupation stability, or housing conditions, which should be considered in future research to disentangle the independent effects of nutrition and socioeconomic context on oral health behaviors.

*Study limitations* should be acknowledged. First, the cross-sectional design precludes causal inference. Second, the OHRQoL outcome was highly skewed, reducing sensitivity for detecting associations and preventing valid multivariable regression modeling. Third, potential residual confounding by socioeconomic status (income, housing, access to dental services) was not fully addressed. Fourth, some assessments relied on parental report (brushing duration, assistance) or observation without inter-rater reliability checks (technique, grip type), introducing possible measurement bias. Finally, the study was conducted only in Bandung, limiting generalizability to other regions.

Despite these limitations, this study highlights important associations between nutritional status, caries burden, and oral health behaviors in preschool children. Future research should incorporate more balanced OHRQoL measures, broader socioeconomic variables, and validated observational tools. Integration of oral health promotion into national stunting reduction strategies—through Posyandu, Puskesmas, and early childhood education centers—remains a promising avenue for preventive action.

## Conclusion

This study highlights significant associations between nutritional status and children’s oral health behaviors, particularly toothbrushing frequency and technique. Stunted children exhibited poorer toothbrushing ability and a higher burden of dental caries compared to their non-stunted peers. Although parental assistance, brushing duration, and grip type were not significantly associated with nutritional status, these aspects may still influence the development of long-term oral health habits.

The highly skewed distribution of OHRQoL responses limited the detection of associations at the global score level; however, item-level analyses revealed meaningful differences between stunted and non-stunted children in several domains, underscoring the potential impact of oral conditions on daily functioning even in early childhood.

These findings emphasize the importance of integrating oral health promotion into early childhood nutrition and stunting prevention programs. Collaborative approaches involving Posyandu, Puskesmas, and early childhood education centers may offer effective platforms for promoting both general and oral health among nutritionally vulnerable populations.

## Supplementary Information

Supplementary Material 1.

Supplementary Material 2.

## Data Availability

The datasets used and/or analysed during the current study are available from the corresponding author, Prof Arlette Suzy Setiawan (email: arlette.puspa@unpad.ac.id), on reasonable request.

## Abbreviations

PCPQ-16: Pediatric Quality of Life Questionnaire for Oral Health
OHRQoL: Oral Health–Related Quality of Life
OHIS: Oral Hygiene Index Simplified
def-t: Decayed, extracted, and filled teeth (primary)
SD: Standard Deviation
