# Supplementary material for: Toothbrushing ability, caries burden, and oral health–related quality of life among stunted preschool children in Bandung-Indonesia
Source: BMC Oral Health. 2025 Dec 29;25:1958. doi: 10.1186/s12903-025-07307-1 (PMC12750588; doi:10.1186/s12903-025-07307-1)
Supplement: Supplementary file 1 — Supplementary Material 1. [file 12903_2025_7307_MOESM1_ESM.docx]

**Assessment and Categorization of Toothbrushing Ability**

| **Item** | **Definition** | **Scoring** | **Assessment** | **Conclusion** |
| --- | --- | --- | --- | --- |
| Toothbrushing frequency | **Toothbrushing frequency** refers to how often a child brushes their teeth within a typical day or week. | **Less than once per day** (e.g., a few times per week) = Score 1  **Once per day** = Score 2  **Twice or more per day** = Score 3 | Questionnaire | **Assessment and Categorization of Toothbrushing Ability**  Children's toothbrushing ability was assessed through five indicators: frequency of toothbrushing (parent-reported),, parental assistance during toothbrusing (parent-reported),, duration of toothbrushing (parent-reported), brushing technique (observed), toothbrush grip (observed). Each indicator was scored on a scale from 0 to 3 based on established criteria. The total score ranged from 0 to 15.  Based on the total score, toothbrushing ability was categorized as follows:   - **Excellent (13–15 points):** Child brushes independently, demonstrates effective technique, and maintains good oral health. - **Good (10–12 points):** Child has adequate brushing ability, with some aspects requiring minor improvement. - **Fair (7–9 points):** Brushing skills are developing, but the child still requires parental supervision and guidance. - **Poor (≤6 points):** Brushing ability is limited, with low hygiene effectiveness; intervention and education are needed.   This composite scoring approach provides a comprehensive evaluation of the child's oral hygiene behavior and outcomes, combining both behavioral and clinical aspects. |
| Parental assistance during toothbrushing | **Parental assistance during toothbrushing**refers to the degree to which a parent or caregiver supports or helps the child during the act of brushing their teeth. This indicator reflects the child's level of independence in performing oral hygiene and the caregiver’s involvement in ensuring effective brushing, particularly in young children who may lack the motor skills or awareness to brush adequately on their own.   - **Effective assistance (active parental involvement)**: Parent or caregiver assists or supervises brushing to ensure proper technique and duration. - **Guided independence**: Child brushes independently but under supervision or with occasional parental correction - **Ineffective or inconsistent assistance**: Parent sometimes helps, but brushing is often skipped or poorly performed - **No brushing activity**: Child does not brush and no parental support is provided | **Effective assistance (active parental involvement)** = Score 3  **Guided independence** = Score 2  Ineffective or I**nconsistent assistance** = Score 1  **No brushing activity** = Score 0 | Questionnaire |  |
| Duration of Toothbrushing | **Duration of toothbrushing** refers to the amount of time a child spends actively brushing their teeth during a single session. This indicator reflects both the child’s attention span and consistency in performing oral hygiene. Adequate duration is important for effective plaque removal, especially in young children who may have less precise brushing techniques.  The American Dental Association (ADA) and pediatric dental guidelines recommend **a minimum of 2 minutes** of brushing per session, twice daily, even for young children | ≥ 2 minutes = Score 3  About 1.5 minutes = Score 2  About 1 minute = Score 1  < 1 minute or not brushed = Score 0 | Questionnaire and observation |  |
| Toothbrushing technique | The movement pattern used by the child when brushing their teeth was observed and classified as circular, horizontal, vertical, or a combination of techniques.   - Circular (Fones method): Small, gentle circular motions; recommended for children due to its effectiveness and safety for gums. Combination: A mix of two or more techniques (e.g., circular and vertical); acceptable if circular is dominant. - Vertical: Up-and-down brushing strokes; less effective and may be too aggressive if not performed properly. - Horizontal: Back-and-forth scrubbing motion; not recommended due to risk of abrasion and poor plaque removal. | Circular = Score 3 Combination = Score 2  Vertical = Score 1  Horizontal = Score 0 | Questionnaire and observation |  |
| Toothbrush grip | **Toothbrush grip** refers to the way a child holds the toothbrush during brushing. It reflects the child’s fine motor development and ability to control the brush. Grips are categorized based on ergonomic and developmental patterns.   - Precision grip: Child holds the toothbrush using fingertips with refined control (similar to holding a pencil) - Oblique grip: Toothbrush is held diagonally with partial palm and fingers — allows some control. - Power grip: Full fist grip, often used by younger children — strong but less precise. - Spoon grip / Others: Unusual or incorrect grip (e.g., holding like a spoon or unstable manner). | Precision grip = Score 3  Oblique grip = Score 2  Power grip = Score 1  Spoon grip / Others = Score 0 | Questionnaire and observation |  |
| **Caries index** | **Caries index** is a clinical measure used to assess the presence and extent of dental caries (tooth decay) in a child. For primary dentition, the most commonly used index is the **deft index**, which includes:   - **d = decayed teeth** - **e = extracted teeth due to caries** - **f = filled teeth** | No Caries (deft 0) = Score 3  Low Caries (deft 1-2) = Score 2  Moderate Caries (deft 3-5) = Score 1  Severe Caries (deft > 6) = Score 0 | Clinical examination |  |
| Oral hygiene | **Oral hygiene:** The condition of oral cleanliness, assessed using OHI-S, which reflects the presence of plaque and calculus on tooth surfaces. | Good oral hygiene (OHI-S 0.0 – 1.2) = Score 3  Fair oral hygiene (OHI-S 1.3 – 3.0) = Score 2  Poor oral hygiene (OHI-S 3.1-6.0) = Score 1 | Clinical examination |  |
