# Supplementary material for: Toothbrushing ability, caries burden, and oral health–related quality of life among stunted preschool children in Bandung-Indonesia
Source: BMC Oral Health. 2025 Dec 29;25:1958. doi: 10.1186/s12903-025-07307-1 (PMC12750588; doi:10.1186/s12903-025-07307-1)
Supplement: Supplementary file 2 — Supplementary Material 2. [file 12903_2025_7307_MOESM2_ESM.docx]

Table Suppl 1. Frequency Distribution of P-CPQ Item Responses by Nutritional Status (Stunted vs Non-Stunted Children)

| **Domain** | **Item** | **Respons Category** | **Stunted (n=278)** | | **Non-stunted (n=276)** | | **p-value** |
| --- | --- | --- | --- | --- | --- | --- | --- |
|  |  |  | **f** | **%** | **f** | **%** |  |
| Oral symptoms | Toothache, lip, jaw, or mouth discomfort | Never | 168 | 60.4 | 106 | 38.4 | 0.000 |
|  |  | Rarely | 62 | 22.3 | 12 | 4.3 |  |
|  |  | Occasionally | 38 | 13.7 | 158 | 57.3 |  |
|  |  | Sometimes | 5 | 1.8 | 0 | 0 |  |
|  |  | Often | 5 | 1.8 | 0 | 0 |  |
|  | Bleeding gums | Never | 258 | 92.8 | 273 | 98.9 | 0.008 |
|  |  | Rarely | 12 | 4.3 | 3 | 1.1 |  |
|  |  | Occasionally | 4 | 1.4 | 0 | 0.0 |  |
|  |  | Sometimes | 1 | 0.4 | 0 | 0.0 |  |
|  |  | Often | 3 | 1.1 | 0 | 0.0 |  |
|  | Bad breath or halitosis | Never | 170 | 61.2 | 181 | 65.6 | 0.001 |
|  |  | Rarely | 65 | 23.4 | 77 | 27.9 |  |
|  |  | Occasionally | 31 | 11.2 | 18 | 6.5 |  |
|  |  | Sometimes | 12 | 4.3 | 0 | 0.0 |  |
|  |  | Often | 0 | 0.0 | 0 | 0.0 |  |
|  | Food getting stuck between teeth | Never | 81 | 29.1 | 29 | 10.5 | 0.000 |
|  |  | Rarely | 71 | 25.5 | 35 | 12.7 |  |
|  |  | Occasionally | 86 | 30.9 | 138 | 50.0 |  |
|  |  | Sometimes | 23 | 8.3 | 69 | 25.0 |  |
|  |  | Often | 17 | 6.1 | 5 | 1.8 |  |
| Function limitation | Difficulty biting or chewing hard food | Never | 171 | 61.5 | 243 | 88.0 | 0.000 |
|  |  | Rarely | 25 | 9.0 | 4 | 1.4 |  |
|  |  | Occasionally | 65 | 23.4 | 29 | 10.5 |  |
|  |  | Sometimes | 3 | 1.1 | 0 | 0.0 |  |
|  |  | Often | 14 | 5.0 | 0 | 0.0 |  |
|  | Taking longer to eat meat than usual | Never | 144 | 51.8 | 125 | 45.3 | 0.000 |
|  |  | Rarely | 39 | 14.0 | 1 | 0.4 |  |
|  |  | Occasionally | 41 | 14.7 | 84 | 30.4 |  |
|  |  | Sometimes | 34 | 12.2 | 54 | 19.6 |  |
|  |  | Often | 20 | 7.2 | 12 | 4.3 |  |
|  | Difficulty eating/drinking hot/cold foods | Never | 228 | 82.0 | 268 | 97.1 | 0.000 |
|  |  | Rarely | 21 | 7.6 | 6 | 2.2 |  |
|  |  | Occasionally | 12 | 4.3 | 2 | 0.7 |  |
|  |  | Sometimes | 13 | 4.7 | 0 | 0.0 |  |
|  |  | Often | 4 | 1.4 | 0 | 0.0 |  |
|  | Sleep disturbance due to toothache | Never | 249 | 89.6 | 276 | 100.0 | 0.000 |
|  |  | Rarely | 9 | 3.2 | 0 | 0.0 |  |
|  |  | Occasionally | 13 | 4.7 | 0 | 0.0 |  |
|  |  | Sometimes | 7 | 2.5 | 0 | 0.0 |  |
|  |  | Often | 0 | 0.0 | 0 | 0.0 |  |
| Emotional wellbeing | Frustration/irritation due to dental problems | Never | 240 | 86.3 | 190 | 68.8 | 0.000 |
|  |  | Rarely | 14 | 5.0 | 0 | 0.0 |  |
|  |  | Occasionally | 17 | 6.1 | 86 | 31.2 |  |
|  |  | Sometimes | 7 | 2.5 | 0 | 0.0 |  |
|  |  | Often | 0 | 0.0 | 0 | 0.0 |  |
|  | Embarrassment due to dental conditions | Never | 261 | 93.9 | 276 | 100.0 | 0.000 |
|  |  | Rarely | 12 | 4.3 | 0 | 0.0 |  |
|  |  | Occasionally | 5 | 1.8 | 0 | 0.0 |  |
|  |  | Sometimes | 0 | 0.0 | 0 | 0.0 |  |
|  |  | Often | 0 | 0.0 | 0 | 0.0 |  |
|  | Anxiety/fear of teeth being different from other children | Never | 268 | 96.4 | 270 | 97.8 | 0.078 |
|  |  | Rarely | 5 | 1.8 | 6 | 2.2 |  |
|  |  | Occasionally | 5 | 1.8 | 0 | 0.0 |  |
|  |  | Sometimes | 0 | 0.0 | 0 | 0.0 |  |
|  |  | Often | 0 | 0.0 | 0 | 0.0 |  |
| Social Wellbeing | Reluctance to speak | Never | 268 | 96.4 | 276 | 100.0 | 0.006 |
|  |  | Rarely | 5 | 1.8 | 0 | 0.0 |  |
|  |  | Occasionally | 5 | 1.8 | 0 | 0.0 |  |
|  |  | Sometimes | 0 | 0.0 | 0 | 0.0 |  |
|  |  | Often | 0 | 0.0 | 0 | 0.0 |  |
|  | Refusal to smile/laugh | Never | 274 | 98.6 | 276 | 100.0 | 0.135 |
|  |  | Rarely | 2 | 0.7 | 0 | 0.0 |  |
|  |  | Occasionally | 2 | 0.7 | 0 | 0.0 |  |
|  |  | Sometimes | 0 | 0.0 | 0 | 0.0 |  |
|  |  | Often | 0 | 0.0 | 0 | 0.0 |  |
|  | Absenteeism from school | Never | 124 | 44.6 | 161 | 58.3 | 0.000 |
|  |  | Rarely | 16 | 5.8 | 3 | 1.1 |  |
|  |  | Occasionally | 12 | 4.3 | 0 | 0.0 |  |
|  |  | Sometimes | 4 | 1.4 | 0 | 0.0 |  |
|  |  | Often | 9 | 3.2 | 0 | 0.0 |  |
|  |  | N/A | 113 | 40.6 | 112 | 40.6 |  |
|  | Difficulty concentrating in learning | Never | 274 | 98.6 | 275 | 99.6 | 0.369 |
|  |  | Rarely | 3 | 1.1 | 1 | 0.4 |  |
|  |  | Occasionally | 1 | 0.4 | 0 | 0.0 |  |
|  |  | Sometimes | 0 | 0.0 | 0 | 0.0 |  |
|  |  | Often | 0 | 0.0 | 0 | 0.0 |  |
|  | Refusal to play | Never | 278 | 100 | 276 | 100 | - |
|  |  | Rarely | 0 | 0.0 | 0 | 0.0 |  |
|  |  | Occasionally | 0 | 0.0 | 0 | 0.0 |  |
|  |  | Sometimes | 0 | 0.0 | 0 | 0.0 |  |
|  |  | Often | 0 | 0.0 | 0 | 0.0 |  |

**Table Suppl 2. Frequency distribution of answers to the toothbrushing ability questionnaire**

| **Question** | **Respond** | **Stunting** | | **Non-stunting** | | **p-value** |
| --- | --- | --- | --- | --- | --- | --- |
|  |  | **f** | **%** | **f** | **%** |  |
| How often does your child brush their teeth? | Never brushes | 9 | 3.2 | 15 | 5.4 | 0.046 |
|  | 1-2 times per week | 23 | 8.3 | 34 | 12.3 |  |
|  | Once a day | 71 | 25.5 | 84 | 30.4 |  |
|  | Twice a day or more | 175 | 62.9 | 143 | 51.8 |  |
| Does your child still need your help when brushing their teeth? | No brushing activity | 3 | 1.1 | 5 | 1.8 | 0.804 |
|  | Parent sometimes helps, but brushing is often skipped or poorly performed | 20 | 7.2 | 24 | 8.7 |  |
|  | Child brushes independently but under supervision or with occasional parental correction | 90 | 32.4 | 88 | 31.9 |  |
|  | Parent or caregiver assists or supervises brushing to ensure proper technique and duration | 165 | 59.4 | 159 | 57.6 |  |
| How long does it take your child to brush their teeth? | < 1 min or not brushed | 4 | 1.4 | 8 | 2.9 | 0.338 |
|  | About 1 min | 87 | 31.3 | 99 | 35.9 |  |
|  | About 1.5 min | 123 | 44.2 | 106 | 38.4 |  |
|  | > 2 minutes | 64 | 23.0 | 63 | 22.8 |  |
| What tooth brushing technique does your child use when brushing their teeth? | Horizontal | 7 | 2.5 | 15 | 5.4 | 0.027 |
|  | Vertical | 142 | 51.1 | 164 | 59.4 |  |
|  | Combination | 94 | 33.8 | 73 | 26.4 |  |
|  | Circular | 35 | 12.6 | 24 | 8.7 |  |
| How does your child hold a toothbrush? | Spoon grip/others | 40 | 14.4 | 44 | 15.9 | 0.625 |
|  | Power grip | 119 | 42.8 | 129 | 46.7 |  |
|  | Oblique grip | 108 | 38.8 | 93 | 33.7 |  |
|  | Precision | 11 | 4.0 | 10 | 3.6 |  |
